# Hypodermic Cathartics

**Published:** 1899-01

**Authors:** E. Stanton Muir

**Affiliations:** Instructor in Materia Medica and Pharmacy, Veterinary Department, University of Pennsylvania


### HYPODERMIC CATHARTICS.

By E. Stanton Muir, Ph.G., V.M.D.,
INSTRUCTOR IN MATERIA MEDICA AND PHARMACY, VETERINARY DEPARTMENT, UNIVERSITY OF PENNSYLVANIA

The question of the hypodermic administration of medicine to the horse is one of great importance to the veterinarian, as there are times, and many of them, too, when quick action from a drug is desired, and at those times the condition and actions of the animal are such as to preclude the possibility of administering our remedial agents without great danger to the practitioner or his assistant. With these points in view, an interesting and instructive course of experiments was conducted at the Veterinary Department of the University of Pennsylvania during the course of 1897 and 1898, with hypodermic cathartics on the horse. These experiments brought out many points which, if not all new to the profession, are of sufficient interest to warrant their being written up for their benefit.

The drugs used were Physostigmine sulphate (eserine), Arecoline hydrobromate, and Barium chloride. Each of these drugs has its champions, but the experiments to be presented to you this evening for discussion were fairly made, and every point bearing directly or indirectly on the subject was noted, and every effort was put forth to make the course as complete as possible, in order that the record should be of value to the profession.

Statistics, to say the least, are uninteresting, but when the subject is one of as much importance to the profession as that under consideration, the desire to increase our knowledge, if nothing else, will make it interesting.

*Physostigmine sulphate* is the sulphate of an alkaloid obtained from the fruit of Physostigma venenosum, one of the leguminosæ —a climbing plant having a woody stem, and is found growing on and attached to trees and shrubs. The fruit, from which the alkaloid is obtained, is about the size and shape of a large horse-bean, being about one and one-half inches in length by three-quarter inches wide, and having a deep hilum running the entire length of the fruit.

*Arecoline hydrobromate* is a salt of the alkaloid arecoline, and is obtained from the fruit of Areca catechu, a plant indigenous to the East Indies and belonging to the palmaceæ. Its average size is somewhat smaller than that of a pigeon's egg, being cone-shaped and concave at the point of attachment to the stem.

*Barium chloride* is a salt obtained by treating barium carbonate with hydrochloric acid; it is permanent in the air, white, translucent in color, having a disagreeable, bitter taste, and crystallizes in the form of flat, tabular crystals. It is soluble in two and one-half times its weight of cold water, and in its own weight of boiling water. According to Lauder Brunton, the action of barium chloride in increasing the contracture of muscle is almost as great as that of veratrine.

I wish here to express my thanks to Dr. W. W. Martin and Dr. John Pearson for valuable assistance in the practical part of this work.

### PHYSOSTIGMINE SULPHATE.

*Experiment No.* 1 was on a gray gelding, thirteen years old, 16½ hands high, in fair condition; had been standing in a stable for some months; fed on bran, oats, and hay. The animal was suffering from osteoporosis, and weighed 1205 pounds. Temperature 37.4° C.; pulse normal; respirations 9; heart 46 and impact strong; peristalsis and feces normal.

Physostigmine sulphate (Merck's) was used. At 12.04 P.M. 0.1 (gr. 1½) was dissolved in 4 c.c. (ʒj) water and injected into the subcutaneous tissue covering the mastoido humeralis. At 12.14 (ten minutes) pulse rose to 66, small and full; animal was pawing, and peristaltic action increased. At 12.20 colicky, very restless, muscular tremors, starting anterior and becoming general at 12.25 (five minutes), 12.26 (twenty-two minutes), tail was elevated, showing signs of a desire to defecate; muscular twitchings less marked, abdominal sounds becoming more moist. 12.42 (thirty-eight minutes), first defecation, which was normal. 1.06 (sixty-two minutes), tenesmus pronounced; constant desire to defecate; 1.30, pulse 42, soft and full, respirations 10, heart strong. 2.20 (two hours and sixteen minutes since injection), eleven passages, making ten since 12.42, or in one hour and thirty-eight minutes; mucous running from anus. 3 to 3.30, marked muscular weakness and drowsiness; no feces were voided from 2.20 until 5 P.M., but an almost constant passage of mucus; weight of feces and mucus voided during experiment, ten pounds.

*Prominent Symptoms.*—Slight colicky pain, increased peristaltic action, with an increase in the flow of mucus in alimentary canal; muscular weakness, apparently starting from point of injection; increased number of defecations, feces gradually becoming softer. These symptoms lasted over five hours.

*Experiment No.* 2.—This animal, a gray gelding, was eight years old, 15½ hands high, weighed 800 pounds, and also had osteoporosis in an advanced stage; was poorly nourished; temperature 37.7° C.; pulse 60, full and soft; heart in fair condition; peristaltic movements normal; feces softer than normal, owing to the fact of his having been fed on bran and hay only previous to the time of the experiment; 0.1 (1.5 gr.) of physostigmine sulphate dissolved in 2. (32 ♏.) of water was injected directly into the jugular at 2 38 P.M. At 2.45, muscular twitchings started, accompanied by slight colicky pain. The first defecation, which was quite soft, was at 2.49, or eleven minutes after injection of the drug. At 2.55 the heart became quite rapid and the animal showed decided colicky symptoms without any increase in muscular symptoms. The second time feces were voided was at 2.55 (seventeen minutes after administration of drug). At 3.25 the pulse was full, soft, and 52. From 2.55 up to 4 P.M. there were ten passages, gradually becoming softer, until 4.32, when the last defecation, which was liquid, took place. After the medicine had commenced to act tenesmus was marked all the time up to 5 P.M., and there was an almost constant flow of mucus from the anus. At 5.20 P.M. the heart was almost normal in its action, and other symptoms were abated. Weight of feces voided during experiment, twenty pounds, or an average of about one and one-quarter pounds at each defecation.

*Prominent symptoms* were almost identical with those noticed in Experiment No. 1, the difference in the two experiments being that in No. 1 the drug was administered subcutaneously and the first defecation took place in thirty-eight minutes after it was given, while in No. 2 the drug was given intravenously and acted in eleven minutes, the dose in each case being the same, 0.1 (1½ gr.). The symptoms also passed off in a quicker time; two hours and forty-two minutes after giving the drug the animal was to all appearances the same as previous to the injection. The amount of feces, you will notice, was double the weight of those passed by animal No. 1.

*Experiment No.* 3.—The animal, a gray gelding, used in this experiment was the one used in Experiment No. 1, three days previous. He was in good condition, and his appetite was good; peristalsis normal and feces normal. Heart 48, strong and full; pulse good. Temperature 37.7° C. (100° F.). In this case 0.2 (3 grs.) of Physostigmine sulphate were dissolved in 4 c.c. (ʒj) water and injected subcutaneously. You will note that this is double the dose used in Experiments Nos. 1 and 2. The injection was made at 10.33 A.M., and at 11.05 the horse had the first passage, which was about of normal consistence; probably the balls were somewhat smaller and harder than normal.

At 11.22 he had a second passage, somewhat softer, and the balls were coated with mucus. The heart up to this time did not change, but at 11.29 it became accelerated, and at the same time the horse began to show signs of pain by pawing. At 11.55 the sixth passage occurred, together with the constant passage of flatus and mucus. Three quarts of oats were given at 12 M., and at 12.08 a second subcutaneous injection of 0.2 (3 gr.) of the drug dissolved in 4 c.c. (ʒj) of water was administered. At 12.10 P.M. he had the seventh passage, but unaccompanied by pain. At 12.15, however, he began to be restless, and was pawing. The eighth defecation was made at 12.16, the anus was relaxed and flabby, and the horse for the first time refused to eat. Muscular weakness and trembling did not occur during this experiment until 12.25, when he also showed signs of great distress, distended nostrils, short, deep, jerky respirations, and a general pained look, all of which gave evidence of the distressed condition of the animal. At 12 34 he passed feces for the thirteenth time, the respirations being hard, labored, and jerky, accompanied by great general weakness. The twenty-third defecation occurred at 1.18, and was liquid and accompanied with considerable tenesmus and passage of mucus. The twenty-fifth and last passage occurred at 1.38, the animal becoming easier until 4 P.M., when he regained his normal condition. Weight of feces, seventeen pounds. The line of symptoms in this experiment differed slightly from those noted in Experiments Nos. 1 and 2, but I attributed this to the fact that, having experimented on this animal three days previously with the same drug, he had to a certain extent become accustomed to its use.

*Experiment No.* 4.—Gray gelding used in Experiment No. 2 three days previous. General condition poor; had been fed on bran and hay; would eat little of the latter on account of having osteoporosis. Pulse 60 and normal; heart impact, good and strong. Temperature 37.4° C. (99.6° F.).

At 10.44 A.M. injected 0.2 (3 grs.) Physostigmine sulphate, dissolved in 3 c.c. (♏ xlviii) water, into the jugular; in 7 minutes, 10.51, he showed decided colicky pains, with elevation of the upper lip. At 10.56, or in twelve minutes from the time of administration, he had a copious passage of a normal consistence for this horse; general muscular tremors with a second defecation were noted at 10.58. At 11.03 there was the fourth passage, accompanied by considerable tenesmus. The horse urinated at 11.05, and we note here that it was only one of two cases of urination during any of the experiments with physostigmine. The respiration became stertorous and labored at 11.10, and at 11.12 there was the eighth passage of soft feces, with evidences of abdominal pain. The heart continued normal, as did the temperature. In fact, we did not notice any change in temperature in any experiment with this drug. 11.42, twenty-first defecation, the rose of the anus protruding from the constant straining and there was a constant passage of mucus, with occasional outbursts of flatus. At the twenty-fifth passage of feces, at 11.52, the pains had subsided, the feces were copious, and almost liquid; considerable tenesmus and passage of flatus were also noticed.

At 12 M. gave him two quarts of bran, which he ate very slowly. The thirty-sixth passage was noted at 12.58, and consisted of feces, flatus, and mucus, which latter was very watery. Borborygmi increased slowly since 10.50. At 1.08 he urinated freely, and the abnormal symptoms were subsiding. The last and thirty-eighth defecation occurred at 1.30, and was quite liquid. The heart's action was strong at all times during this experiment, but was sometimes slightly quickened.

The condition of the animal was about normal in all respects at 4 P.M., and the weight of feces passed was twenty-eight pounds. This animal, like the one used in Experiment No. 3, may have been granted a certain amount of immunity on account of having been experimented on with the same drug three days previous.

*Experiment No.* 20.—It has been held by some therapeutists and practitioners that after a solution of eserine has stood for some time, and acquired the characteristic red color, it will not have its full therapeutic effect. To prove or disprove this assertion we concluded to have one more experiment with this drug. The solution used had stood exposed to the light for over a week, and had become of a deep red color. The subcutaneous injection of 0.1 (1½ grs.) was made at 12.30 P.M. on a gray gelding, eight years old, 15¼ hands high, and weighing 750 pounds; the general condition of the animal was poor. Temperature normal; pulse soft and regular; respirations normal; heart rather weak. At 12.41 (eleven minutes after administration) the animal became uneasy and passed feces. Up to 12.59, twenty-nine minutes after administering the drug, the horse had eight good-sized passages, and continued having more or less abdominal pain. From this time up to 1.35 he had four more passages, each one being softer and accompanied with more mucus. The weight of the feces voided was not taken, as the experiment proved beyond a doubt that the solution loses none of its activity by standing. I might add that I have used solutions in my private practice that were over a month old, and have never failed to get the purgative action.

From these experiments I think the following deductions can be made:

1. Physostigmine is an active cathartic on the horse.

2. It does not cause sufficient pain to render it unsafe to use in all cases where its use is indicated.

3. It not only increases peristalsis, but also causes an increase in the secretion of mucus, thus rendering the feces more and more liquid.

4. Its action does not last, even when given in large doses, over four or five hours.

5. It causes no change in temperature, and only a slight quickening of the heart's action.

6. The quicker the action comes on the more copious are the discharges and the sooner does the action cease.

7. No bad results follow its use—*i. e.*, when given in therapeutic doses.

8. Muscular weakness always accompanies its action—*i. e.*, it is a muscle-poison.

9. It may safely be given in 0.2 (3 gr.) doses either subcutaneously or intravenously. It will act much slower, however, in 0.1 (1½ gr.) doses.

10. It has no sudorific properties.

11. It not only acts quicker, but causes a larger quantity of feces to be voided when given intravenously.

### Arecoline Hydrobromate.

Much controversy has been held over the merits and demerits of the various hypodermic cathartics in their use in colic, etc., and for this reason we will at the end of this paper give a chart comparing the action of the three drugs under consideration. We will now, however, note the results obtained in a series of experiments with Arecoline hydrobromate (Merck's) on the horse. The grated fruit has been used as a vermifuge for the dog for years. Its use on the horse is, however, more recent, it having been used in intestinal impaction, colics, and laminitis.

*Experiment No.* 9.—The first experiment (No. 9) with this drug was on a gray gelding in fair condition; pulse 60, strong and full; heart strong and regular; respirations 10; temperature normal. At 11, 0.05 ($\frac{3}{4}$ gr.) dissolved in water was injected into the jugular. In two minutes the animal was trembling, respirations were labored, and the flow of saliva was profuse. The respirations quickly rose from 10 to 28. At 11.04, four minutes from the time of injection, the pulse dropped to 40 and was soft. The first defecation was at 11.04. Up to 11.08 the animal had defecated four times and the flow of saliva had increased. At 11.09 the pulse had dropped to 36. At 11.19 he had had twelve passages, gradually becoming a little softer, and there was at that time incontinence of urine.

The breathing became slow, deep, and stertorous at 11.31, and the pulse continued at 36. At 1.38 or two hours and thirty-eight minutes after making the injection, the action had ceased, flow of saliva had ceased, and the animal was eating hay as if nothing had occurred to disturb him.

Another intravenous injection of 0.05 ($\frac{3}{4}$ gr.) dissolved in water was then administered, and in two minutes ptyalism was again pronounced, and the animal was retching just as does a dog that has overloaded his stomach and wishes to get rid of its contents. At 1.45 the animal attempted, unsuccessfully, to defecate. Incontinence of urine started again at 1.50, at which time he had a moist passage. From that time until 1.59 three more watery passages were noted, and up to 2 P.M. there were ptyalism and a constant dripping of urine. About this time all abnormal symptoms ceased, and the animal started to eat. Colicky symptoms were pronounced all the way through this experiment.

*Prominent symptoms* were profuse ptyalism, purgation, feces not becoming soft so quickly as with physostigmine; colicky pains more severe; incontinence of urine marked; amount of feces passed not excessive, as was the case with physostigmine.

*Experiment No.* 10.—Bay gelding, eight years old, 15½ hands high, weight 1025 pounds. Temperature normal; heart strong; impact good; pulse 48, full and strong. This animal was poor in flesh, but otherwise in good condition. At 11.15 a subcutaneous injection of 0.05 ($\frac{3}{4}$ gr.) of Arecoline hydrobromate dissolved in water was given. The first sign of ptyalism was noted at 11.21. The animal having had no passages and only a slightly increased flow of saliva, a second injection of 0.05 ($\frac{3}{4}$ gr.) of the drug was given at 11.34, nineteen minutes after the first injection. At

12.19, forty-five minutes from second injection, there was a passage of feces, normal in quantity, etc.

No other symptoms showing, a third subcutaneous injection of 0.1 ($1\frac{1}{2}$ grs.) was made at 12.48. At 1.15 profuse ptyalism started. At 1.25 the animal defecated, the feces being normal in all respects. Colicky symptoms developed at 1.45. At 2 he had the third passage since beginning the experiment; still normal condition of feces.

At 2.53, all symptoms having abated, a fourth injection of 0.2 (3 grs.) of the drug was given. In four minutes the flow of saliva was profuse. Until 3.04 he was in extreme pain, lying down, rolling, and evincing all the symptoms that accompany a bad case of colic. Sweating started at 3.12, and he voided the fourth quantity of feces. The feces passed at 3.18 were becoming somewhat more moist, and sweating was increased until at 3.21 he did not have a dry hair on his body, and the sweat was dripping from his belly in streams. At 3.30 laid down, passed considerable flatus, arose at 3.42, and had the seventh defecation, which was moist, after which he almost immediately laid down. At 3.49 he arose and seemed easier, but laid down again at 3.51, with head extended, ears flabby, and a pained facial expression. At 4.30 was still colicky. At 5 the symptoms gradually subsided, the improvement being slow, he not being all right until 10 P.M. The quantity of feces voided was small, not being considered sufficient to warrant the use of the drug, in colic at least, considering the amount of pain it caused.

*Prominent symptoms* did not differ materially from those seen in Experiment No. 9. Of course, in this experiment four doses 0.4 (6 grs.) of the drug were used, extending over a period of three hours and thirty-eight minutes, while in Experiment No. 9 but 0.1 ($1\frac{1}{2}$ grs.) was administered in two doses two hours and forty-two minutes apart. This accounts for the increased severity of the symptoms.

*Experiment No.* 28.—The animal used for this experiment was a bay gelding in rather poor condition, probably as good as the average huckster's horse, and was quite old, $15\frac{3}{4}$ hands high, weight 900 pounds. Temperature normal; pulse 52 and weak; respirations 6; heart regular; impact weak. An intravenous injection of 0.1 ($1\frac{1}{2}$ grs.) of Arecoline hydrobromate was made at 11.12; almost immediately (11.13) there was trembling, loss of coördination, and ptyalism produced. The respirations became labored at once; at 11.14 became stertorous. First defecation at

11.18, six minutes after making the injection. The balls were small and hard. At 11.25 there were marked contractions of the abdominal muscles, with attempts to vomit. At 11.34 the pulse was 54, the heart rapid and strong, the horse becoming easier, and ptyalism ceasing twenty-two minutes after giving the drug. From this time the symptoms gradually abated, until at 1.30 he was all right. At that hour we gave a second intravenous injection of 0.1 ($1\frac{1}{2}$ grs.), and from that time until 4.10 colicky pains, ptyalism, and four passages of feces of normal consistence were noted. All symptoms having passed off at 4.30, the feces were weighed and found to be eight pounds and four ounces.

*Prominent symptoms* were the same as in the other experiments: Constant abdominal pains, attempt to vomit, passage of a moderately increased amount of feces of a normal consistence, stertorous breathing, profuse ptyalism, loss of coördination, and incontinence of urine.

*Experiment No.* 29.—Animal was a bay gelding, 12 years old, $15\frac{3}{4}$ hands high, weighs 1000 pounds. Pulse 60 and full; heart strong; respirations 14; general condition good.

At 11.10 injected a solution containing 0.2 (3 grs.) into the jugular. Trembling, salivation, and loss of coördination were noted at 11.12, two minutes after administering the drug. The first defecation was three minutes after giving the drug at 11.13; condition of feces normal. At 11.16 the symptoms were less pronounced. Sweating began at 11.20, and was mostly posterior; pulse full; heart 72; 11.36, venous pulse, ptyalism ceased, animal easier; 12.01, urinated; quantity and condition normal. Another injection was given at 1.47 of 0.1 ($1\frac{1}{2}$ grs.) of the drug, and from that time until 4.10 five passages were noted, the last two being soft, the others normal. All abnormal symptoms had passed off at 4.30, and on weighing the feces we found there were twelve and one-quarter pounds.

*Experiment No.* 30.—This bay gelding was in rather poor condition, was eleven years old, 16 hands high, weighed 1000 pounds. Pulse full and strong; heart 40 and regular; respirations 14. At 11.06 an intravenous injection of 0.2 (3 grs.) of Arecoline hydrobromate was given. In seven minutes there were slight muscular twitchings, and at 11.14 there was a passage of normal feces. At 11.19 ptyalism was profuse, and the third defecation of normal feces was noted. Borborygmi were well marked, and muscular twitchings were severe at 11.21, when he had the fourth passage, the feces being small in quantity and normal in consistence.

Respirations were decidedly labored at 11.26, when the sixth passage of feces occurred. Decided weakness was also noticed at that time. General twitching of the muscles continued, and sweat was dripping from all parts of the body at 11.29, when the seventh defecation occurred. At 11.33 the peculiar contraction of the abdominal muscles was marked, at which time there was a strong venous pulse. Up to 11.56 there were in all twelve defecations, the weight of which was only eight pounds. Sweating, ptyalism, muscular twitching and weakness continued until 12.25, when the hair began to dry. Pronounced abdominal pain continued throughout the experiment.

These experiments seem to prove the following:

1. Arecoline hydrobromate produces profuse ptyalism, but does not increase the secretion of moisture in the alimentary canal to any marked degree.

2. It causes severe abdominal pain.

3. The reduction in temperature is probably due to its sudorific action.

4. It causes the pulse to become slower and softer, and in many cases causes a venous pulse.

5. Its action lasts longer than that of eserine, is more severe, and not so certain.

6. The distress caused by its administration is due partly to the colic it produces and partly to its emetic action.

7. It may with safety be given in 0.02 ($\frac{1}{3}$ gr.) doses and repeated in one hour.

8. It causes incontinence of urine.

(To be continued.)

---

Tariff rates on live animals intended for Cuba after January 1, 1899, viz.: Horses and mares above the standard height, $10; all others, $5; mules, $5; asses, $1; cows, $1; bullocks, calves, and heifers, $1; pigs, $1; sheep, goats, and animals not specifically mentioned, $1.

The recent resolution adopted by the Council of the Royal Agricultural Society prohibiting the exhibition of docked horses in the three-year-old and under classes has been rescinded in response to the protestation of exhibitors and breeders.

Fannie, an old mare of the Conant family, of Mitchell, S. D., recently died at the age of thirty-eight years.

